# Construction of genetic linkage map and identification of a novel major locus for resistance to pine wood nematode in Japanese black pine (*Pinus thunbergii*)

**DOI:** 10.1186/s12870-019-2045-y

**Published:** 2019-10-15

**Authors:** Tomonori Hirao, Koji Matsunaga, Hideki Hirakawa, Kenta Shirasawa, Keiya Isoda, Kentaro Mishima, Miho Tamura, Atsushi Watanabe

**Affiliations:** 1 Forest Bio-research Center, Forestry and Forest Products Research Institute, 3809-1 Ishi, Juo, Hitachi, Ibaraki 319-1301 Japan; 2 Kyushu Regional Breeding Office, Forest Tree Breeding Center, Forestry and Forest Products Research Institute, 2320-5 Suya, Goshi, Kumamoto, 860-0081 Japan; 3 Department of Frontier Research, Kazusa DNA Research Institute, Chiba, 292-0818 Japan; 4 Forest Tree Breeding Center, Forestry and Forest Products Research Institute, 3809-1 Ishi, Juo, Hitachi, Ibaraki 319-1301 Japan; 5 Department of Forest Environmental Sciences, Faculty of Agriculture, Kyushu University, 6-10-1 Hakozaki, Higashi-ku, Fukuoka, 812-8581 Japan

**Keywords:** *Pinus thunbergii*, Pine wood nematode, Pine wood disease, Resistance, Genetic linkage map

## Abstract

**Background:**

Pine wilt disease (PWD), which is caused by the pine wood nematode (PWN) *Bursaphelenchus xylophilus*, is currently the greatest threat to pine forests in Europe and East Asian countries including Japan. Constructing a detailed linkage map of DNA markers and identifying PWD resistance genes/loci lead to improved resistance in *Pinus thunbergii*, as well as other *Pinus* species that are also susceptible to PWD.

**Results:**

A total F_1_ mapping population of 188 individuals derived from a cross between the PWD-resistant *P. thunbergii* varieties ‘Tanabe 54’ (resistant rank 2 to PWD) and ‘Tosashimizu 63’ (resistant rank 4 to PWD) was inoculated with PWN, and was evaluated for disease symptoms. To perform linkage analysis for PWN resistance, a set of three maps was constructed; two parental maps generated using the integrated two-way pseudo-testcross method, and a consensus map with population-type cross-pollination. The linkage map of ‘Tanabe 54’ consisted of 167 loci, and covered 14 linkage groups (LGs), with a total genetic distance of 1214.6 cM. The linkage map of ‘Tosashimizu 63’ consisted of 252 loci, and covered 14 LGs, with a total genetic distance of 1422.1 cM. The integrated consensus map comprised 12 LGs with the basic chromosome number of *P. thunbergii*, and a total genetic distance of 1403.6 cM. Results from quantitative trait loci (QTL) analysis using phenotype data and linkage maps indicated that PWN resistance is controlled by a single dominant allele, which was derived from the ‘Tanabe 54’ female parent. This major QTL was located on linkage group 3 and was designated *PWD1* for *PINE WILT DISEASE 1*.

**Conclusions:**

The *PWD1* locus is a major resistance QTL located on the *Pinus* consensus LG03 that acts in a dominant manner to confer pine wood nematode resistance. Information from the present study will be useful for *P. thunbergii* breeding programs to improve resistance to PWD, and also to help identify susceptibility genes in *Pinus* species.

## Background

Pine wilt disease (PWD), caused by the pine wood nematode (PWN) *Bursaphelenchus xylophilus* [1], occurs when the number of PWN within a pine tree increases to such an extent that water transport through the infected tree is compromised, leading to wilting and eventually death [2, 3]. At present, PWD constitutes the greatest threat to pine forests worldwide [2]. The causal agent of PWD is the PWN, and pine sawyer beetles (*Monochamus* spp.) act as a vector. The PWN has been considered to have originated in North America [4] and is widely distributed in the United States; however, no PWD epidemics have been found and the disease have only occurred in a few exotic pine species [5, 6]. In the areas where it occurs, pine wood nematode is an artificially introduced, invasive pathogen that has caused extensive damage in Japan in 1968, China in 1982, Korea in 1988 and Europe in 1999 [2].

The PWD is a chronic problem in the pine forests (*Pinus thunbergii* Parl. and *P. densiflora* Sieb. & Zucc) of Japan, where approximately 40,000,000 m^3^ of pine forests have been blighted by the PWN since 1978 [7]. As a means of dealing with PWD in Japan, a breeding project to develop resistant pine varieties was started in western Japan in 1978 [8], and related projects have been promoted throughout Japan, excluding Hokkaido Island, as the damage has spread [7, 9]. In the first breeding project from 1978 to 1984, 16 PWD-resistant *P. thunbergii* clones (selection efficiency; 0.1%) were selected from 15,000 candidate trees, and 92 PWD-resistant *P. densiflora* clones (selection efficiency; 0.8%) were selected from 11,000 candidate trees [8]. The selected clones were then reared to develop PWD-resistant *Pinus* varieties.

These varieties were evaluated based on the survival rate of open-pollinated progeny following inoculation with PWN; average rates of survival of openly pollinated progeny from resistant varieties were 51% for *P. thunbergii* and 65% for *P. densiflora*, which is respectively 35 and 18% higher than for unselected populations [10]. The narrow-sense heritability based on the open-pollinated family of 15 out of 16 varieties was estimated to be a maximum of 0.486 in *P. thunbergii*, indicating that resistance (tolerance) is inherited in an additive manner [11]. Furthermore, it was also shown that the number of genetic factors for *P. thunbergii* resistance was 1.96 based on diallel analysis using a full diallel mating design that used three out of 16 varieties including ‘Tanabe 54’ [12]. The findings have shown that about two genetic factors are involved and that resistance is additive; however, the locus for PWD resistance has not been identified using a molecular genetic approach.

Identifying the genetic determinants for PWD resistance is not only critical for the development of PWD-resistant clones in *P. thunbergii*, but would also provide valuable information for screening of resistance loci/genes and breeding strategies in other *Pinus* species. In East Asian countries (China, Korea, and Taiwan), PWD has spread in *Pinus* species such as *P. massoniana* Lamb. and *P. koraiensis* Sieb. & Zucc [13, 14]. In Europe, PWD is also spreading rapidly, causing serious damage in *P. pinaster* Ait. forests [15–19]. Furthermore, *Pinus* forests in Europe and East Asia are thought to be at risk of serious damage due to habitat shifts and the spread of PWD due to climate change [20]. In addition, a variety of factors such as shifts in the insect vectors of PWN, the state of international wood trade, and incomplete phytosanitary treatment may also cause further damage [21]. To cope with PWD, screening, selection and genetic evaluation of candidate individuals or families for resistance or tolerance have been carried out in each *Pinus* species, with the aim of identifying resistant individuals and securing genetic resources [18, 22, 23]. Resistant parents/individuals may be quickly used to promote genetic improvement and more cost-effective breeding if gene-assisted selection (GAS) or marker-assisted selection (MAS) tools are available.

Genetic linkage maps play a major role in genetic analysis and molecular breeding programs, and have been widely used for identification of genetic loci using agronomic traits, such as biological or abiotic stress resistance and yield, which can promote genetic improvement and more cost-effective breeding. In some *Pinus* species, high-density genetic linkage maps have been constructed using genomic simple sequence repeat (SSR), expressed sequence tag (EST)-derived SSR, or single nucleotide polymorphism (SNP) markers [24–27], and linkage analysis and genome-wide association studies (GWAS) for agronomic traits (e.g., growth, wood properties, and abiotic/biotic stress resistance) have already been conducted [27, 28]. The previous genetic linkage map in *P. thunbergii* was mainly constructed by relying on dominant markers, such as random amplification of polymorphic DNA (RAPD) markers to identify loci for resistance to the pine needle gall midge (*Thecodiplosis japonensis*), and consisted of 17 linkage groups spanning 1469.8 cM with 98 markers [29, 30]. However, it is essential to increase the resolution and density of genetic linkage maps using co-dominant markers in order to identify an effective locus for target traits and to develop a marker for MAS.

To our knowledge, this is the first QTL mapping study of PWD resistance. The present study sought to identify loci related to PWD resistance in resistant *P. thunbergii* varieties. The specific aims were: (i) to construct a high-confidence genetic linkage map for *P. thunbergii*; and (ii) to identify loci for PWD resistance in *P. thunbergii* using the constructed genetic linkage map.

## Results

### Evaluation of PWD resistance in the population

We evaluated the phenotypes in an F_1_ population at 56 days post inoculation (dpi) according to the rating scale shown in Table 1. External symptoms occurred at 21 dpi and progressed rapidly until 35 dpi, and disease symptoms were stabilized at 56 dpi (Additional file 1). At 56 dpi, phenotypic scores for PWN resistance showed significant separation into a bimodal distribution with the resistant group (scores 1–2) comprising 50% of all individuals tested, while the susceptible group (scores 3–4) comprised 50% of all individuals tested (Table 2). The phenotypic segregation ratios were not statistically different from 1:1 (*x*^*2*^ = 0.00, *p* = 1.00), and were different from 3:1 (*x*^*2*^ = 62.67, *p* = 0.00). These results indicate that PWD resistance in the F_1_ population is controlled by one major gene. We used the phenotypic scores of the F_1_ population at 56 dpi for the QTL analysis.

**Table 1:** Rating scale for phenotypic assessment of PWD resistance

Phenotype	Score	Symptoms	
Resistant	1	No visible symptoms	
	2	Slight leaf discoloration	
Susceptible	3	Severe discoloration and dry leaves	
	4	Dark-brown stem, large expanding lesions

**Table 2:** Segregation ratio of PWD resistance in ‘Tanabe 54’ x ‘Tosashimizu 63’ F_1_ population following the rating scale of Table 1

Progeny	No. of plants/Phenotypic score				Total	Binary standard^a^		Expected ratio (1:1)*		Expected ratio (3:1)*	
	S1	S2	S3	S4		Resistant	Susceptible	X^2^	*p*-value	X^2^	*p*-value
Tanabe 54′ x ‘Tosashimizu 63’	88	6	2	92	188	94	94	0.00	1.00	62.67	0.00

*Chi-squared and *p*-values (one degree of freedom) are calculated under the assumption of a Mendelian 1:1 and 3:1 segregation ratio

^a^ Resistant and Susceptible are based on S1 + S2 (Resistant) and S2 + S3 (Susceptible)

### Marker polymorphism

Sixty-one (70.11%) of the 87 genomic SSR markers and 28 (13.59%) of the 206 EST-SSR markers were informative for at least one of the mapping parents. In addition to the SSR data, 138 (17.97%) of the 768 SNPs using the Fluidigm 96.96 Dynamic Array Chip and 268 (30.99%) of the 768 SNPs in the GoldenGate Assay were informative for at least one of the mapping parents; thus, the total number of markers used for linkage mapping was 465. Of these, 98 (21.08%) were segregated in both parents: 17 with four alleles (ab × cd); 16 with three alleles (ef × eg); 65 with two alleles (hk × hk); and 140 (30.11%) were heterozygous in only ‘Tanabe 54’ (lm × ll), while 254 (48.82%) were heterozygous in only ‘Tosashimizu 63’ (nn × np) (Table 3). Being bi-allelic, SNPs were scored as lm × ll, nn × np and hk × hk.

**Table 3:** Segregation types for different marker analyses conducted in the F_1_ population derived from resistant variety ‘Tanabe 54’ and resistant variety ‘Tosashimizu 63’

Markers	No. of markers analyzed	Segregation types^a^					No. of markers constructing linkage map^b^
		ab × cd	ef × eg	hk × hk	lm × ll	nn × np	
Genomic SSR	87	17	9	1	18	16	61
EST-SSR	206	–	7	1	7	13	28
Fluidigm SNP	768	–	–	23	36	79	138
GoldenGate SNP	768	–	–	40	79	119	238

^a^ Parental genotypes were coded in accordance with JoinMap 4.1 [31]

^b^ Markers that were mapped to maternal, paternal, or consensus map

### Construction of a genetic linkage map

**Fig. 1:**
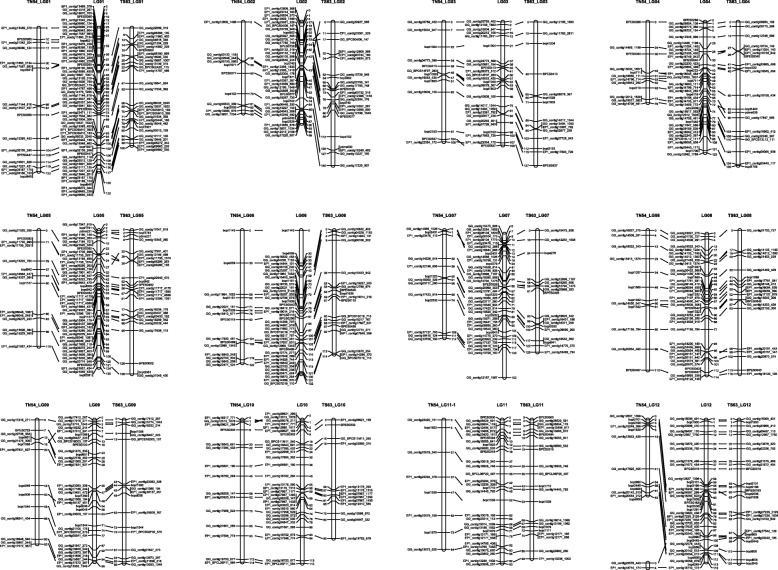
Genetic linkage map of *P. thunbergii.* The map in the middle (LG01-LG12) is the consensus map constructed from the combined dataset. The maps on the left (TN54_LG01-LG12) and right (TS63_LG01-LG12) are the maternal (‘Tanabe 54’) and paternal (‘Tosashimizu 63’) maps, respectively. Homology between these is depicted by lines. Marker positions are indicated in cM. See Additional files 1, 2, 4, and 5 for DNA marker types mapped on linkage maps

Three maps were constructed; the two parental maps generated using the integrated two-way pseudo-testcross method, and an integrated consensus map using JoinMap 4.1 (Fig. 1). The linkage map for ‘Tanabe 54’ consisted of 167 loci, and covered 14 LGs (TN54_LG01 to TN54_LG13), with a total genetic distance of 1214.6 cM. The linkage map for ‘Tosashimizu 63’ consisted of 252 loci, and covered 14 LGs (TS63_LG01 to TS63_LG14), with a total genetic distance of 1422.1 cM. The integrated consensus map comprised 12 LGs with a total genetic distance of 1403.6 cM. The average distances between markers in the maps for ‘Tanabe 54’ and ‘Tosashimizu 63’ were 7.9 cM and 6.2 cM, respectively. Gaps wider than 30 cM were identified in five LGs of ‘Tanabe 54’ (TN54_LG01, 02, 03, 05, and 07) and in five LGs of ‘Tosashimizu 63’ (TS63_LG05, 06, 07, 08, and 10) (Table 4).

**Table 4:** Description of ‘Tanabe54’, ‘Tosashimizu64’ and consensus linkage maps

LG^a^	Tanabe54′ (maternal)						LG	Tosashimizu64’ (paternal)						LG	Consensus					
	gSSR	EST-SSR	SNPs	Length (cM)	Avg. marker distance (cM)	Max. gap (cM)		gSSR	EST-SSR	SNPs	Length (cM)	Avg. marker distance (cM)	Max. gap (cM)		gSSR	EST-SSR	SNPs	Length (cM)	Avg. marker distance (cM)	Max. gap (cM)
01	2	3	13	121.9	7.2	32.1	01	1	1	27	99.8	3.6	14.2	01	3	4	49	122.5	2.2	14.4
02	3	1	7	88.9	8.9	37.7	02	4	2	18	139.5	6.1	26.2	02	6	2	30	98.7	2.7	15.5
03	4	2	7	105.5	8.8	35.1	03	4	2	9	127.2	9.1	27.1	03	4	2	20	109.3	4.4	15.7
04	2	2	8	75.9	6.9	23.9	04	4	1	15	135.0	7.1	23.6	04	6	2	31	123.1	3.3	12.3
05	2	1	12	110.1	7.9	30.1	05	4	2	19	136.0	5.7	31.7	05	6	3	37	130.4	3.0	12.0
06	4	2	9	121.6	8.7	29.7	06	1	3	16	114.0	6.0	30.1	06	4	4	32	131.6	3.4	30.1
07	3	0	10	113.9	9.5	34.7	07	3	1	13	129.4	8.1	32.0	07	5	1	33	152.0	4.0	32.1
08	4	1	7	113.2	10.3	18.8	08	1	2	17	116.2	6.1	33.2	08	4	3	31	114.4	3.1	16.0
09	5	1	8	82.9	6.4	24.9	09	4	0	14	95.6	5.6	29.7	09	6	1	27	94.8	2.9	16.1
10	0	1	14	113.8	8.1	19.5	10	0	1	12	94.9	7.9	36.9	10	0	1	30	114.8	3.8	19.5
11	3	0	5	83.7	12.0	15.9	11	4	3	15	90.3	4.3	13.2	11	4	3	25	93.1	3.0	9.7
12–1	7	0	6	63.2	5.3	27.4	12	10	2	13	120.0	5.0	15.6	12	11	2	24	118.9	3.3	12.6
12–2	0	0	2	4.3	–	–														
13	1	0	5	15.7	3.1	5.0	13	0	0	2	16.7	–	–	–	–	–	–	–	–	–
							14	0	0	2	7.5	–	–	–	–	–	–	–	–	–
Total	40	14	113	1214.6	7.9	25.8	Total	40	20	192	1422.1	6.2	26.1	Total	59	28	369	1403.6	3.3	17.2

^a^ LG is linkage group, and the numbering of linkage groups in the present study was determined by comparison with the *P. taeda* linkage map [引用文献]. The LG12 of ‘Tanabe 54’ consisted of two linkage groups

We compared the constructed linkage map in the present study to the *P. taeda* consensus maps reported recently [24]. The sequences of 397 ESTs mapped on the *P. thunbergii* linkage map were used to identify the numbering of the linkage group and the order of the DNA markers in *P. thunbergii* based on the *P. taeda* linkage map. Of the 397 genes mapped on the *P. thunbergii* linkage map, 109 were identical to *P. taeda* genes, with BLASTn e-values ranging from 10E-14 to 0 (Additional file 2). The relative order of mapped genes on the other linkage maps for two *Pinus* species were highly correlated, *R*^2^ = 0.898–0.999 (Additional file 3), although there were not enough markers to compare the relative order of the mapped genes in some linkage groups (LG04), and clear positional differences were seen with a small number of markers in another linkage group (LG12).

### Identification of a novel locus for PWD resistance

**Fig. 2:**
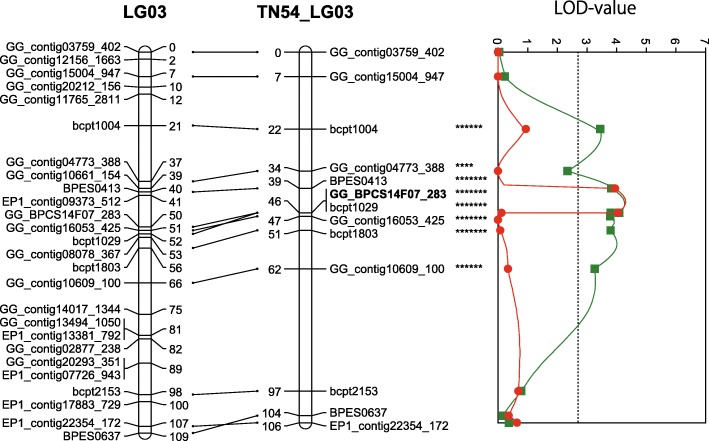
QTL *PWD1* for pine wood disease resistance on linkage group 3. The maps on the left (LG03) and right (TN54_LG03) are the consensus and the maternal (‘Tanabe 54’) maps, respectively. One-LOD support intervals (bars) and diagrams with the LOD values (dashed line) are displayed for interval mapping (line with squares) and MQM analysis (line with circles). The peaks reached a LOD maximum of 4.4, respectively, and explained 10.2% of the variance observed. Marker positions are indicated in cM. The marker used as a cofactor in MQM is highlighted in bold

A QTL analysis performed using a constructed genetic linkage map and phenotypic data from a PWN inoculation test revealed a locus for PWD resistance on LG03 (Fig. 2 and Table 5). This QTL on LG03 was identified in only maternal ‘Tanabe 54’ linkage group 3, and a maximum plateau LOD score value (LOD = 4.27) was detected. The permutation tests with 1000 permutations yielded a LOD score threshold for the PWD resistance of 2.6 at a statistical significance level of α = 0.05, which was used as the threshold to detect QTL for PWD resistance with genome-wide significance. Interval mapping (IM) and Multiple QTL mapping (MQM) mapping confirmed the location of a major QTL explaining up to 9.9% of the total phenotypic variance observed for PWD resistance with a maximum LOD of 4.27 from 38.7 cM to 45.8 cM on LG03. On the other hand, QTL data reanalyzed using an integrated map to reconfirm QTLs detected on the parental linkage maps revealed one major QTL on LG03, and a maximum plateau LOD score value (LOD = 5.51). Permutation tests with 1000 permutations yielded a LOD score threshold for PWD resistance of 4.3 at a statistical significance level of α = 0.05, which was used as the threshold to detect QTL for PWD resistance with genome-wide significance. Interval mapping and MQM mapping confirmed the location of a major QTL explaining up to 12.6% of the total phenotypic variance observed for PWD resistance with a maximum LOD of 5.51 from 41.8 cM to 50.8 cM on LG03. Cofactor selection in MQM mapping selected marker GG_BPCS14F07_283 as a cofactor for this region in all scores.

**Table 5:** Location, significance and confidence interval of QTL identified by MQM in ‘Tanabe 54’ x ‘Tosashimizu 63’ F_1_ population for PWD resistance

Tanabe 54′ linkage map							Integrated map					
LG^a^	LOD threshold-GW^b^	LOD threshold-LG specific	Max LOD	% Var^c^	Nearest marker	QTL confidence interval (cM)	LOD threshold-GW^b^	LOD threshold-LG specific	Max LOD	% Var^c^	Nearest marker	QTL confidence interval (cM)
03	2.6	1.6	4.27	9.9	GG_BPCS14F07_283	38.7–45.8	4.3	2.9	5.51	12.6	GG_BPCS14F07_283	41.8–50.8

^a^ Linkage group

^b^ Genome-wide

^c^ Percentage phenotypic variance explained

Using the non-parametric Kruskal-Wallis (KW) test, PWD resistance was associated with three genomic SSR markers; one EST-derived SSR marker, and two EST-derived SNP markers on LG03 (Table 6). Furthermore, all QTLs on LG03 were explained by additive maternal effects (Table 6).

**Table 6:** Significant markers that segregate with resistance to PWD as identified with Kruskal-Wallis test

Surrounding markers									Mean of genotype classes associated with phase 00^f^				Allelic effects^g^		
Locus^a^	Type of DNA marker	Allele configuration	LG^b^	maternal ‘Tanabe 54’ map position	Integrated map position	K^c^	Signif. KW^d^	Df^e^	ac (ee)	ad (ef)	bc (eg)	bd (fg)	Maternal	Paternal	Interaction
bcpt1004	genomic SSR	<abxcd>	03	22.17	21.12	15.04	******	1	101.6	114.6	82.3	81.7	52.20	−12.40	−13.60
BPES0413	EST-SSR	<efxeg>	03	38.75	39.81	16.71	*******	1	84.1	103.9	78.8	116.3	−57.30	−7.10	17.70
**GG_BPCS14F07_283**	EST-derived SNP	<lmxll>	03	45.82	49.82	17.82	*******	1	–	–	–	–	–	–	–
bcpt1029	genomic SSR	<abxcd>	03	46.36	51.98	16.62	*******	1	121.4	98.4	81.5	80.7	57.60	23.80	22.20
GG_contig16053_425	EST-derived SNP	<lmxll>	03	47.44	51.09	16.60	*******	1	–	–	–	–	–	–	–
bcpt1803	genomic SSR	<abxcd>	03	50.69	56.39	16.62	*******	1	83.8	78.8	121.4	98.4	−57.20	28.00	−18.00

^a^ Bold indicates the nearest marker of QTL identified by MQM

^b^ Linkage group

^c^ Kruskal–Wallis analysis (K*) test regarded as nonparametric equivalent of one-way analysis of variance (Van Ooijen 2004)

^d^ *P*-values are designated as ****** = *p*<0.001; ******* = *p* < 0.0001

^e^ Degree of freedom

^f^ Represents four genotypic segregating classes (ac, ad, bc, bd) of a cross of two heterozygous parents <ab x cd>, being ab as maternal and cd as paternal alleles

^g^ Additive effects calculated as [(ac + ad) - (bc + bd)] for male, [(ac + bc) - (ad + bd)], and [(ac + bd) - (ad + bc)] for interaction effects. The bigger the number, the more significant effects from the class

## Discussion

In Japan, projects for resistance breeding have produced 183 resistant clones of *P. thunbergii* and 246 resistant clones of *P. densiflora* since 1978. Although heritability for resistance traits has been verified using open seedlings of several resistant families [11], the loci involved have not been identified.

This study represents the first attempt to detect the region for PWN resistance. An F_1_ mapping population derived from a resistant variety cross (‘Tanabe 54’ x ‘Tosashimizu 63’) was used to map the PWN resistance locus. Results from the nematode inoculation test performed on F_1_ individuals indicated that PWN resistance is controlled by a single dominant allele, which is derived from the ‘Tanabe 54’ female parent. This major QTL for resistance was designated *PWD1* for *PINE WILT DISEASE 1*.

### Construction of genetic linkage map

Genetic linkage maps are important in genetic research and breeding for mapping desirable traits and identifying QTL and numerous genetic markers required for constructing linkage maps. In a previous study, 17 linkage groups spanning 1469.8 cM with an average marker density of 15.0 cM were constructed mainly relying on dominant markers such as RAPD markers to identify resistance to the pine needle gall midge (*T. japonensis*) in *P. thunbergii*. The present linkage map converged onto 12 linkage groups spanning a distance of 1403.6 cM with an average marker density of 3.3 cM, which defined the positions of 59 polymorphic SSR markers and 369 SNP markers, and were close to the lengths of previously constructed linkage maps.

Furthermore, the development and use of EST-derived SSR and SNP markers in the present study made it possible to construct a more informative genetic linkage map of *P. thunbergii* by comparison with the information from *P. taeda*, which has the most dense and accurate genetic information among the Pinaceae taxa studied to date [24]. In some linkage groups, there were not enough markers to compare the relative order of the mapped genes (LG04), but clear positional differences were observed with a small number of markers in the linkage group (LG12). In order to examine the conservation of synteny and chromosome rearrangements between the *P. taeda* and *P. thunbergii* genomes for the all linkage groups, including linkage group 4 and 12 (LG04 and LG12), more markers need to be employed in the future. However, the genetic linkage map constructed in this study provided sufficient information to estimate the position of major QTLs for resistance and a number of candidate genes to be targeted in the future.

Paternal and maternal linkage maps constructed using a double pseudo-test cross strategy revealed shorter linkage groups for the maternal map (1214.6 cM) than those for the paternal map (1422.1 cM). The difference in length might be due to differences in the number of informative SNP markers between the overall linkage groups on the maternal (113 SNPs) and paternal (192 SNPs) maps. In addition, the length of LG04 and LG12 showed a noteworthy inconsistency between the maternal and paternal maps. To explore whether this is related to sex, population size or sequencing errors, additional markers need to be developed and more research is required.

### QTL analysis for PWD resistance

In the present study, disease severity was evaluated using four degrees of severity, and was converted to a binary standard, such as mortality, which provided reliable data for QTL calculations. For phenotype evaluation, assessment of external wilting symptoms after artificial inoculation is the most commonly used method with regard to evaluating susceptibility and resistance in host pines, and it was revealed that PWD resistance is a heritable trait by genetic analysis of a gene family based on mortality evaluation (alive and dead) [9, 11, 32]. In the present study, PWD resistance in the F_1_ population was found to be controlled by one major gene as indicated by mortality due to disease severity, and this finding was supported by actual QTL analysis.

The QTL analysis using phenotypic values for PWD resistance in the F_1_ population succeeded in detecting a major *PWD1* locus for PWD resistance on the constructed map of LG03 (Fig. 2 and Table 5), and corresponded to ‘Tanabe 54’ maternal segregation (Fig. 2, Tables 5 and 6). Although the initial hypothesis did not include the detection of a major *PWD1* locus related to PWD resistance only in ‘Tanabe 54’, it is a very interesting result demonstrating the heritability of PWD resistance. In a previous study, the heritability of resistance traits in resistant varieties was evaluated based on survival rates of open seedlings, and it was shown that the survival rate of open-pollinated progeny in ‘Tanabe 54’ and ‘Tosashimizu 63’ was 47.5 and 61.1%, respectively, while that of susceptible pines was 12.5% [33]. Furthermore, narrow-sense heritability estimates based on survival data of the open-pollinated family of 15 out of 16 resistant varieties was a maximum of 0.486, indicating that resistance (tolerance) would be inherited in an additive manner [11], and the number of genetic factors for *P. thunbergii* resistance was 1.96 [12]. The knowledge obtained from previous studies and the results of this study confirmed that ‘Tanabe 54’ has a heterozygous allele involved in PWD resistance, and this locus is a major dominant that exhibits additive effects. We therefore consider that one of the two genetic factors related to PWD resistance reported in a previous study [12] has been identified in this study.

The locus for PWD resistance was detected in ‘Tanabe 54’, but not ‘Tosashimizu 63’. One possible explanation is that ‘Tosashimizu 63’ is a recessive homozygote at the other locus involved in PWD resistance, but the effect cannot be detected in the present crossing. In fact, the locus detected in ‘Tanabe 54’ contributes little to the phenotypic trait (percentage of explained phenotypic variance for the trait by marker). Furthermore, it is also possible that the alleles for PWD resistance in ‘Tosashimizu 63’ had been affected by epistatic interactions by alleles of ‘Tanabe 54’, as well as possibility of having a QTL involving multiple loci rather than a major gene. To identify the resistance locus of ‘Tosashimizu 63’, it will be necessary to cross ‘Tosashimizu 63’ again with susceptible F_1_ individuals obtained by crossing ‘Tanabe 54’ and ‘Tosashimizu 63’. However, these susceptible individuals will need to be cloned because they will be lost in the inoculation test, and will require a great deal of time to reach the age at which mating is possible. It will be necessary to cultivate new materials and perform inoculation tests in order to identify the resistance locus of ‘Tosashimizu 63’ in the future.

The resistance locus (*rhg1*-b) for soybean cyst nematode (*Heterodera glycines*) is recognized as an additive and incompletely dominant gene, and the locus is mediated by copy number variation of three genes within a 31-kb span [34–36]. The locus *PWD1,* which contributes little to the phenotypic trait, might show genetic behavior and mutations (CNV) similar to the soybean cyst nematode resistance (*rhg-1b*).

By comparison with recently updated *P. taeda* consensus genetic maps [24], we were able to determine that the *PWD1* locus is located on the *Pinus* consensus LG03. The relatively highly conserved gene order (collinearity) of the *P. thunbergii* and *P. taeda* linkage maps facilitates the identification of candidate genes or genomic regions for *PWD1* in *P. thunbergii* and related *Pinus* species. Genetic and genomic studies of white pine blister rust resistance in *P. flexilis* and *P. lambertiana* have identified several major resistance genes (*Cr1, Cr2, Cr4*) that are anchored on the *Pinus* consensus genetic map by comparison with *P. taeda* genetic maps [37–40]. A candidate gene for PWD resistance might similarly be identified by comparison with the high density linkage map and genomic information for *P. taeda* [25, 40–43]. At the present stage, the QTL region (41.8–50.8 cM) detected in *P. thunbergii* corresponds to the region from 52.1 cM to 117.37 cM on the linkage map of *P. taeda*, which contains tens of genes. Based on this genomic information from *P. taeda*, it may be possible to discover further candidate regions and genes by searching for orthologous genes and DNA markers in *P. thunbergii*.

The pine wilt disease caused by the PWN poses a serious threat to pine forests [20, 44]. In Asia (Japan, South Korea, mainland China, and Taiwan) and Europe (Portugal and Spain), several *Pinus* species (*P. thunbergii*, *P. densiflora*, *P. koraiensis* and *P. massoniana* from Asia, and *P. sylvestris* and *P. pinaster* from Europe) showing susceptibility to PWN have already been severely affected by PWD. Several other *Pinus* species are also at risk of serious damage due to future climate change [20]. Furthermore, not only climate change, but factors such as changes in the insect vectors of PWN, the importation of infested wood material via international trade, and incomplete phytosanitary treatment may further increase the damage [21]. As one of a long-lasting approach to address the PWD problem, breeding programs are currently underway to establish PWD-resistant varieties. The linkage map and PWD resistance genes/loci identified in the present study will provide an opportunity to improve resistance and establish a foundation for further genetic research in *P. thunbergii* and other *Pinus* species that are susceptible to PWD.

In this study, by using multiple genotyping systems, we constructed a base map for *P. thunbergii* that can serve as the basis for a genetic approach to resolving the susceptibility of *Pinus* species to PWD. In addition, this is the first report to identify one of the major loci contributing to PWN resistance in *Pinus* species. Further research needs to be undertaken to develop more markers using DNA microarrays or next-generation sequencing (NGS) and to construct a high density linkage map for *P. thunbergii*. On the other hand, phenotypic evaluation methods for PWD resistance need to be improved to detect more QTL contributing to PWD resistance. To date, genotyping systems using DNA microarray or NGS, a DNA microarray that targets tens of thousands of DNA polymorphisms, and next-generation sequencers have become available for conifer species and high density linkage maps have been constructed [24, 25, 45, 46]. Furthermore, for phenotyping, chlorophyll fluorescence and oleoresin flow have been used to evaluate tolerance to PWN infection in *P. pinaster*, and the physiological condition of pines to PWN has been evaluated [18]. By applying these techniques and methods to other resistant families and resistant *P. thunbergii* populations in the future, it will be possible to advance the identification of loci that contribute to resistance traits, and to realize GAS and MAS in resistance breeding to PWD.

## Conclusions

In this study, we updated the *P. thunbergii* linkage map, which had been constructed with only dominant DNA markers, by using SSR markers and co-dominant markers derived from ESTs. This was facilitated by a comparison with the previously constructed linkage map information for *P. taeda*, which has the highest marker density among Pinaceae. Furthermore, QTL analysis of the F_1_ mapping population, which was conducted using the constructed genetic linkage map and phenotypic data from a PWN inoculation test, revealed a locus for PWD resistance on LG03. This QTL on LG03 was identified in only the maternal ‘Tanabe 54’ linkage group 3, which is a major QTL for resistance.

## Methods

### Plant material

Sixteen resistant varieties of *P. thunbergii* were selected for the first breeding program and were ranked with regard to resistance (levels 1–5) based on the survival rate of openly pollinated progeny following PWN inoculation; higher survival rates are thought to indicate greater resistance [33]. An F_1_ population of 188 individuals was derived from a single cross between two resistant varieties: a female parent ‘Tanabe 54’ and a male parent ‘Tosashimizu 63’ at the Kyushu Regional Breeding Office, Forest Products Research Institute, Forest Tree Breeding Center (FFPRI-FTBC) in Kumamoto, Japan. The female parent ‘Tanabe 54’ had a lower resistance level (level 2), and the male parent ‘Tosashimizu 63’ had a higher resistance level (level 4). Progeny were seeded in March 2013 and planted in a field at the Forest Products Research Institute, Forest Tree Breeding Center (FFPRI-FTBC) in Ibaraki, Japan in April 2014. Seedlings were planted at a spacing of 30 cm × 30 cm. Total genomic DNA of the F_1_ population was extracted from leaves using a DNeasy Plant Mini Kit (Qiagen, Hilden, Germany) and subjected to polymorphism analysis, as described below.

### Artificial inoculation and phenotyping

Inoculation with PWN was conducted on July 23, 2014. The PWN used in this study was the Ka-4 isolate, which has been used in PWD resistance breeding projects since 2003. For inoculation, the main stem was shaved with a knife at 5 cm from the ground to expose cambium cells, and 3000 nematodes suspended in 50 μl of sterile water were injected into the shaved area. Disease symptoms were evaluated every 7 days from 14 dpi to 56 dpi according to the rating scale shown in Table 1.

### Genomic SSR genotyping

A total of 87 genomic DNA-derived SSR markers (accession numbers: LC416800-LC416869), including 17 markers identified in previous studies [47–50], were analyzed using genomic DNA from ‘Tanabe 54’ and ‘Tosashimizu 63’, and informative SSR markers that showed heterozygous patterns in either the female or male parent were used to genotype 188 individuals in the F_1_ mapping population (Additional file 4). Multiplex PCR with three or four SSR primer pairs was performed using a Multiplex PCR Kit (Qiagen), with 2× QIAGEN multiplex PCR master mix, 0.25 μM of each primer pair, and 40 ng of genomic DNA in a total volume of 10 μl. Amplification was performed on a Veriti thermal cycler (Thermo Fisher Scientific, Waltham, MA, USA) using an initial denaturation step of 95 °C for 15 min, followed by 30 cycles of denaturation at 94 °C for 30 s, annealing at 57 °C for 1.5 min, and extension at 72 °C for 1 min, with a final extension step of 60 °C for 30 min. The PCR products (1 μl) were mixed with 0.2 μl GeneScan 500 LIZ size standard (Thermo Fisher Scientific) and 9.8 μl of Hi-Di formamide (Thermo Fisher Scientific) prior to electrophoresis. The length of amplified fragments was analyzed on an ABI 3130xl sequencer (Thermo Fisher Scientific) and alleles were scored with GeneMapper v5.0 software (Thermo Fisher Scientific).

### Development of EST-derived SSR markers and genotyping

PureLink Plant RNA Reagent (Thermo Fisher Scientific) was used to extract total RNA from needles and stems of *P. thunbergii* ‘Namikata 37’. cDNA libraries were constructed for each organ and then sequencing analysis and data processing were performed as described previously [51]. Microsatellites or SSR motifs (a minimum length of 15 bp with minimum repetitions for di-, tri-, and tetra-nucleotides) were searched based on high-quality reads that comprised > 50 bp of contiguous sequence, and 756 candidate SSR markers without duplication were obtained (accession numbers: HX995015-HX995770). Primer pairs were designed against the flanking sequences of each SSR, as described previously [51]. Genomic DNA from 16 *P. thunbergii* varieties was analyzed for the 756 SSR markers. The 206 informative SSR markers that showed polymorphic patterns were used to genotype the 188 F_1_ progeny (Additional file 5). The PCR and genotyping analyses were as described above for genomic SSR genotyping.

### Development of EST-derived SNP markers and genotyping

For construction of the reference transcriptome, we extracted total RNA from the stem of a resistant variety, *P. thunbergii* ‘Namikata 73’, and a susceptible variety (plus-tree), *P. thunbergii* ‘Kataura 1’, inoculated with PWN Ka-4 isolate at eight time points (1, 3, 6, and 12 h post-inoculation, and 1, 2, 3, and 7 dpi). Total RNA was isolated using an RNeasy Plant Mini kit (Qiagen), and the quality of total RNA was assessed via an Agilent Bioanalyzer 2100 system (Agilent Technologies, Palo Alto, CA). cDNA was synthesized from a mixture of RNA samples by nebulization, adaptor ligation and emulsion PCR, and the 1/2 plate was sequenced using a Roche 454-FLX Life Sciences sequencer (Roche/454 Life Sciences, Branford, CT). All sequence data produced from the 454-FLX were deposited in the DDBJ Sequence Read Archive [DDBJ: DRA000531].

Sequences from the Roche 454-FLX system were trimmed of adapter sequences and poly(A/T) sequences using the cutadapt tool, and low-quality sequences and short sequences (< 100 bp) were removed. The Roche 454-FLX reads and Sanger reads obtained to develop EST-SSR above mentioned markers were assembled using Newbler version 2.6, resulting in a unigene set comprising 22,066 contigs and 1286 singletons. A total of 23,352 unigenes were annotated with BLASTX analysis using the NCBI-nr, PGI database, and KEGG GENES database (Additional file 5). All assembled data produced from 454-FLX and Sanger sequencing were deposited in the DDBJ, Transcriptome Shotgun Assembly division [DDBJ: IADK01000001-IADK01023202]; sequences that overlapped with EST-SSR markers gave priority to accession numbers already given (see Additional file 6).

For SNP discovery, resequencing to the reference sequences was performed for five resistant clones and three susceptible clones using the Illumina HiSeq 2500 platform (Illumina, Branford, CT). RNA was extracted from needles and stems prior to nematode inoculation and at 3 days post-inoculation with nematodes, and mixed for each clone. RNA extraction and quality checks followed the same method used for EST library construction. Using a TruSeq RNA Sample Prep kit ver.2 (Illumina), cDNA synthesis from an RNA sample from each organ, nebulization, adaptor ligation (including index tagging for individual recognition), bridge PCR and paired-end sequencing were performed on the Illumina HiSeq 2500 platform. Reads sequenced on the Illumina HiSeq system were also trimmed of adapter sequences and poly(A/T) by cutadapt. Then, the reads of each library were mapped to reference sequences by BWA [31] and SNPs were identified using SAMtools software [52] with default settings.

For SNP genotyping, we used on two independent SNP assay platforms: an array of 768 SNPs on Illumina’s GoldenGate platform and KBiosciences’ KASPar assay platform. The 768 candidate genes and SNPs supplied on each platform were selected by gene expression profiling between resistant and susceptible clones (Hirao et al. unpublished), and special attention was paid to select only one SNP per differentially expressed transcript. For Illumina’s GoldenGate platform, the 768 selected SNPs were designed with the custom oligonucleotide pooled assay (OPA; Illumina Inc., San Diego, CA) containing the allele-specific and locus-specific oligos for use in the Illumina GoldenGate assay. Genotyping of the SNP markers was carried out using the 768-OPA and Illumina’s BeadArray Express Reader according to the standard manufacturer’s protocol. Automatic allele calling for each locus was inferred with the GenomeStudio Software (Illumina). The list of SNPs and their flanking regions that constituted the OPA are presented in Additional file 7.

For the other SNP assay platform, genotyping of SNPs was performed by competitive allele-specific polymerase chain reaction KASPar chemistry (KBiosciences Ltd., Hoddesdon, UK) using the Fluidigm (Fluidigm Corp., San Francisco, CA) 96.96 dynamic array according to the standard manufacturer’s protocol. End-point fluorescent images of the chip were measured with the EP1 reader (Fluidigm Corp.) and plotted on two axes, and genotypes based on EP1 measurements were assessed using Fluidigm SNP Genotyping Analysis software (Fluidigm, 2011). The list of SNPs and their flanking regions for a total of 768 SNP markers (8 marker panels constituting 96 SNPs) are presented in Additional file 8.

### Linkage map construction

Linkage analysis was conducted using JoinMap 4.1 software [53] with population type cross-pollination (CP) [54]. To construct an integrated map of ‘Tanabe 54 ‘and ‘Tosashimizu 63’, all segregating markers that showed polymorphism in at least one parent were used in the JoinMap configurations for CP mode (ab × cd, lm × ll, nn × np, ef × eg, and hk × hk). The ratio of marker segregation was calculated by Chi-squared test. Markers showing significantly distorted segregation (*P*-value < 0.001) were excluded from the map construction. Markers were grouped with a minimum logarithm of odds (LOD) score of 5.0 and a recombination frequency of 0.45. A regression mapping algorithm was used to build the linkage map, and map distances were calculated according to the Kosambi mapping function [55]. All other calculation conditions were used at default settings. The number of linkage groups and the order of markers in this study were determined.

A double pseudo-test cross strategy [54] was applied for genetic linkage map construction. The LOD threshold for mapping was set at 4.0 and the recombination frequency at 0.45. The marker configurations ab × cd, lm × ll, and ef × eg were used for the maternal maps ‘Tanabe 54’, and configurations ab × cd, nn × np, and ef × eg were used for the paternal maps ‘Tosashimizu 63’. The marker configuration hk × hk refers to a bi-parental marker with genotypes hh, hk and kk, and hk was coded as the non-informative marker (−-). The genetic linkage map was drawn with MapChart 2.2 software [56], and homologous linkage groups were compared using the software and illustrated so that each integrated map group was between the parental maps.

The numbering of the linkage map and the order of the DNA markers derived from EST in *P. thunbergii* were estimated by identifying highly conserved *Pinus* genes or orthologs by local BLAST analysis (*E values* < 10e-10) with *P. taeda* [24]. Based on the results of BLAST analysis, the number and orientation of the linkage group or the relative order and location of the mapped genes in *P. thunbergii* were determined based on information from the *P. taeda* linkage map.

### QTL analysis

QTL analysis was performed using MapQTL 6.0 software [57]. QTL identification was initially performed using the parental maps of ‘Tanabe 54’ and ‘Tosashimizu 63’ separately; data were then reanalyzed using an integrated map to reconfirm the QTLs detected on the parental linkage maps. To perform the binary trait method in the present QTL analysis, the resistant and susceptible phenotypes were converted to values equal to 0 and 1 (Table 2), respectively, and two different methods were used. First, interval mapping (IM) at 1 cM intervals was carried out to detect QTLs. The genome-wide and LG-specific LOD (logarithm of the odds) thresholds for each QTL were calculated using a permutation test with 1000 repetitions at *P* < 0.05 (5%). Closely flanking markers were then selected as cofactors and multiple QTL mapping (MQM) performed. Second, the non-parametric KW test module was performed for traits to confirm the significance of the marker nearest the detected QTL. An allelic effect for the QTL was calculated in MapQTL6 based on the SSR genotypes significantly detected by KW using the formula proposed by Knott et al. [58].

## Supplementary information

**Additional file 1: Figure S1.** The phenotypes in an F1 population before inoculation and from 14 days post-inoculation (dpi) of PWN until 56 dpi. Full view of individual numbers 1 to 96.The phenotypes in an F1 population before inoculation and from 14 days post-inoculation (dpi) of PWN until 56 dpi. Full view of individual numbers 97 to 198.

**Additional file 2: Table S1.** Correspondence of orthologous markers mapped on *P. thunbergii* and *P. taeda* linkage groups.

**Additional file 3: Table S2.** Syntenic relationship between *Pinus thunbergii* linkage group and *P. taeda* linkage group. *P. taeda* genetic linkage map data were based on a previous report [25].

**Additional file 4: Table S3.** Details of 87 genomic DNA-derived SSR markers in *Pinus thunbergii*.

**Additional file 5: Table S4.** Summary of BLAST search results of ESTs derived from Roche 454-FLX and Sanger reads assembly. Accession ID of 454 and Sanger data is as for the Transcriptome Shotgun Assembly (TSA) division of the DDBJ database.

**Additional file 6: Table S5.** Details of 206 EST-SSR markers derived from 756 non-redundant ESTs in *Pinus thunbergii*.

**Additional file 7: Table S6.** Characteristics of 768 SNPs of *Pinus thunbergii* on Illumina’s GoldenGate platform. Locus name, LG position, target SNP and sequence flanking the queried SNPs (in brackets) are described.

**Additional file 8: Table S7.** Primer information for 768 SNPs of *Pinus thunbergii* for BioMark 96.96 Dynamic Array (Fluidigm) using KASPar assays. Locus name, LG position, target SNP and sequence flanking the queried SNPs (in brackets) are described. Furthermore, sequences for allele-specific primers (ASP1 and ASP2), locus-specific primer (LSP), and specific target amplification primer (STA) are also described.

## Data Availability

All data generated or analyzed during this study are included in this published article and its Additional information files.

## Abbreviations

EST: Expressed sequence tag
LG: Linkage group
LOD: Logarithm of the odds
MAS: Marker-assisted selection
QTL: Quantitative trait locus
SNP: Single nucleotide polymorphism
SSR: Simple sequence repeat
