# Supplementary figures and images for: Construction of genetic linkage map and identification of a novel major locus for resistance to pine wood nematode in Japanese black pine (Pinus thunbergii)
Source: BMC Plant Biol. 2019 Oct 15;19:424. doi: 10.1186/s12870-019-2045-y (PMC6792208; doi:10.1186/s12870-019-2045-y)

## Slide 1
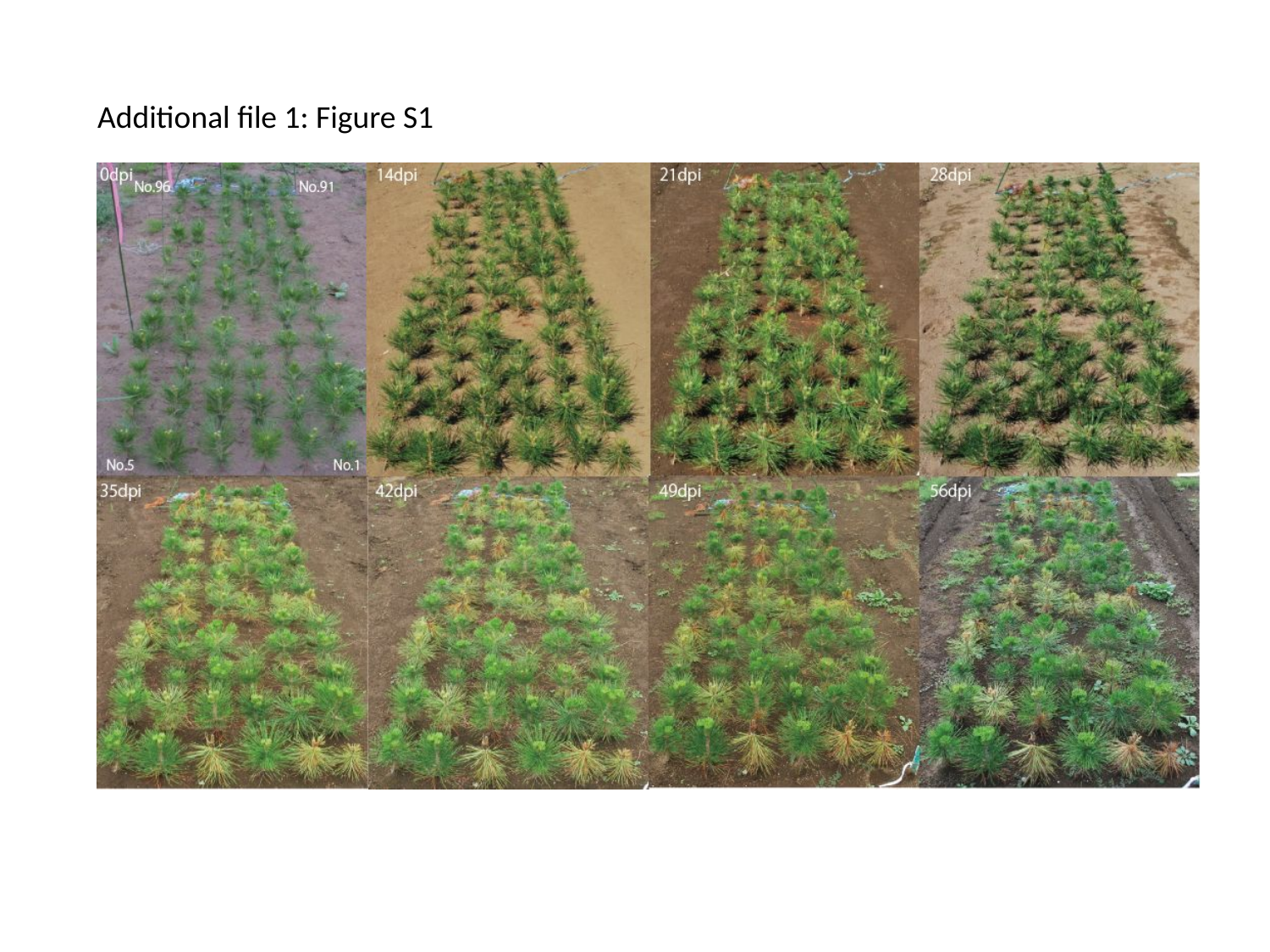

Additional file 1: Figure S1

## Slide 2
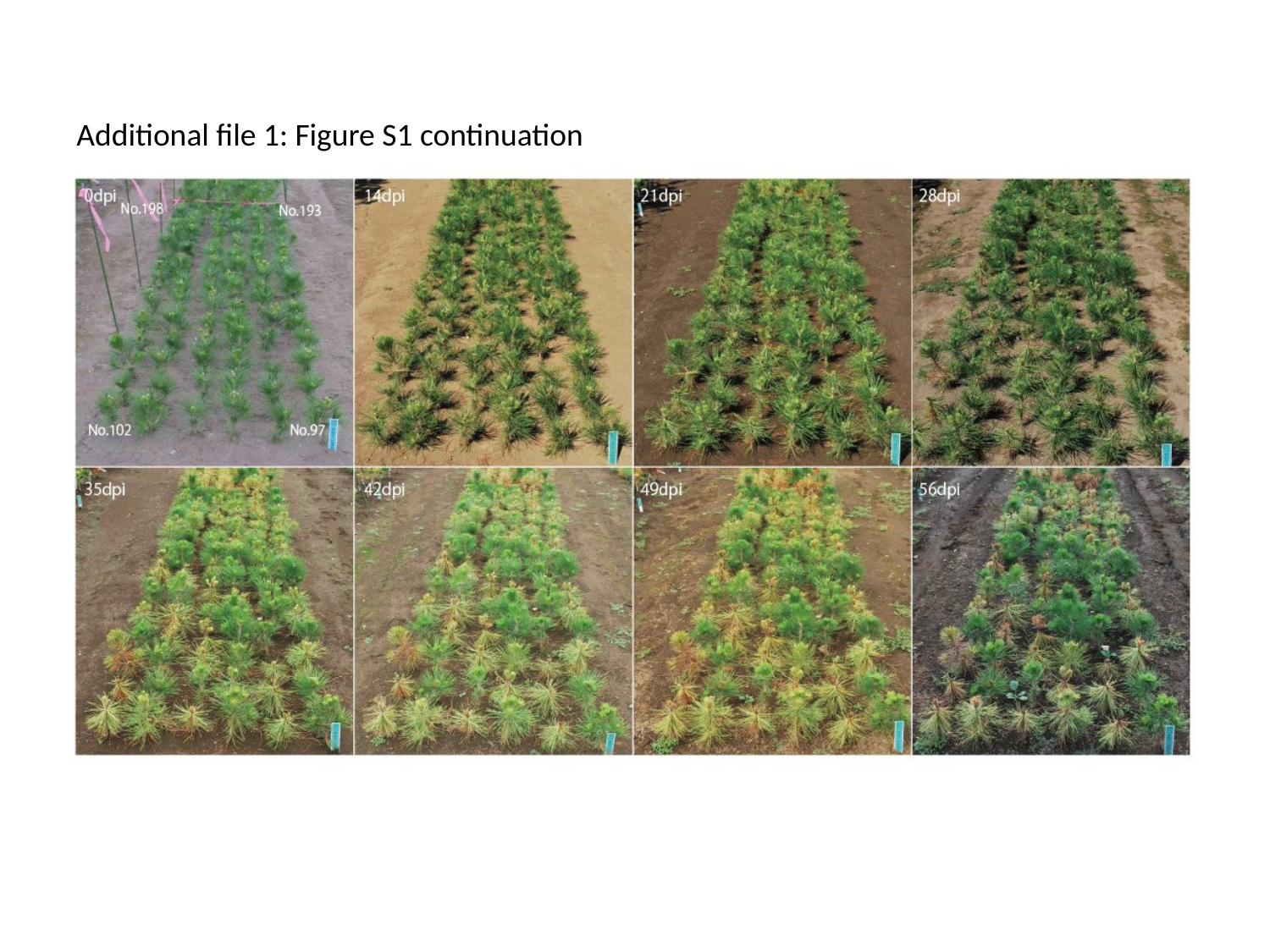

Additional file 1: Figure S1 continuation

Supplement: Supplementary file 1 — Additional file 1: Figure S1. The phenotypes in an F1 population before inoculation and from 14 days post-inoculation (dpi) of PWN until 56 dpi. Full view of individual numbers 1 to 96.The phenotypes in an F1 population before inoculation and from 14 days post-inoculation (dpi) of PWN until 56 dpi. Full view of individual numbers 97 to 198. [file 12870_2019_2045_MOESM1_ESM.pptx]
